# Microbiota-induced regulatory T cells associate with *FUT2*-dependent susceptibility to rotavirus gastroenteritis

**DOI:** 10.3389/fmicb.2023.1123803

**Published:** 2023-02-27

**Authors:** Emmanuelle Godefroy, Laure Barbé, Béatrice Le Moullac-Vaidye, Jézabel Rocher, Adrien Breiman, Sébastien Leuillet, Denis Mariat, Jean-Marc Chatel, Nathalie Ruvoën-Clouet, Thomas Carton, Francine Jotereau, Jacques Le Pendu

**Affiliations:** ^1^Inserm, CNRS, Immunology and New Concepts in ImmunoTherapy, INCIT, UMR 1303/EMR6001, Nantes Université, Nantes, France; ^2^CHU de Nantes, Nantes, France; ^3^Biofortis SAS, Saint-Herblain, France; ^4^INRAE, AgroParisTech, UMR1319, MICALIS, Université Paris Saclay, Jouy en Josas, France; ^5^ONIRIS, Ecole Nationale Vétérinaire, Agroalimentaire et de l'Alimentation, Nantes, France

**Keywords:** inflammatory diseases, *FUT2*, fucosylation, histo-blood group antigens, rotavirus, antibodies, regulatory T cells, *Faecalibacterium prausnitzii*

## Abstract

The FUT2 α1,2fucosyltransferase contributes to the synthesis of fucosylated glycans used as attachment factors by several pathogens, including noroviruses and rotaviruses, that can induce life-threatening gastroenteritis in young children. *FUT2* genetic polymorphisms impairing fucosylation are strongly associated with resistance to dominant strains of both noroviruses and rotaviruses. Interestingly, the wild-type allele associated with viral gastroenteritis susceptibility inversely appears to be protective against several inflammatory or autoimmune diseases for yet unclear reasons, although a *FUT2* influence on microbiota composition has been observed. Here, we studied a cohort of young healthy adults and showed that the wild-type *FUT2* allele was associated with the presence of anti-RVA antibodies, either neutralizing antibodies or serum IgA, confirming its association with the risk of RVA gastroenteritis. Strikingly, it was also associated with the frequency of gut microbiota-induced regulatory T cells (Tregs), so-called DP8α Tregs, albeit only in individuals who had anti-RVA neutralizing antibodies or high titers of anti-RVA IgAs. DP8α Tregs specifically recognize the human symbiont *Faecalibacterium prausnitzii*, which strongly supports their induction by this anti-inflammatory bacterium. The proportion of *F. prausnitzii* in feces was also associated with the *FUT2* wild-type allele. These observations link the *FUT2* genotype with the risk of RVA gastroenteritis, the microbiota and microbiota-induced DP8α Treg cells, suggesting that the anti-RVA immune response might involve an induction/expansion of these T lymphocytes later providing a balanced immunological state that confers protection against inflammatory diseases.

## Introduction

Epithelial cell surfaces are lined by a thick layer of glycans called the glycocalyx. The outermost part of the glycocalyx of various epithelial cell types presents inter- and intra-species variability that constitutes the so-called histo-blood group antigens (HBGAs), based on their initial discovery on erythrocytes (Marionneau et al., 2001; Cooling, 2015). These include the ABO and Lewis antigens that are synthesized by sequential addition of several monosaccharides, including fucose residues. The FUT2 α1,2fucosyltransferase is central to the synthesis process of these carbohydrate structures. The *FUT2* gene is highly polymorphic and represents one of the few human genes under frequency-dependent selection, strongly indicative of a major role in relation with environmental factors (Ferrer-Admetlla et al., 2009; Silva et al., 2010). The presence of a functional (or wild-type) *FUT2* allele generates the so-called secretor phenotype that is characterized by the expression of the A, B or H and Lewis^b^ antigens according to the ABO and Lewis phenotypes. *FUT2* mutant null alleles are responsible for the lack of these antigens which characterizes the nonsecretor phenotype (Marionneau et al., 2001). The frequency of the nonsecretor phenotype is quite variable, ranging from over 40% to less than 10% according to human ethnicity (Faden and Schaefer, 2021). Consistent with a role of the *FUT2* gene polymorphism, the secretor/nonsecretor phenotype has been associated with either resistance or susceptibility to several pathogens, most strikingly with noroviruses and rotaviruses that together are responsible for the vast majority of gastroenteritis cases, leading to the death of several hundred thousand young children yearly, the latter occurring mostly in low-income countries (Ramani et al., 2016; Bányai et al., 2018; Troeger et al., 2018; Nordgren and Svensson, 2019; Sharma et al., 2020). The role of *FUT2* polymorphisms in determining the susceptibility to these viruses is rather well understood. Both human noroviruses and rotaviruses use HBGAs as attachment factors to initiate infection in a strain-specific manner (Ruvoën-Clouet et al., 2013; Tan and Jiang, 2014; Schroten et al., 2016; Ramani and Giri, 2019; Tenge et al., 2021). Distinct strains attach to a variable set of glycan motifs defined by HBGA polymorphisms such that individual strains cannot infect every person in the population, consistent with a host-pathogen co-evolution process (Le Pendu et al., 2014). In other words, it results from an arms race in glycan-mediated host–microbe interactions, that involves a frequency-dependent selection, as previously discussed (Le Pendu and Ruvoën-Clouet, 2020).

The *FUT2* gene polymorphism has additionally been associated with a diverse set of inflammatory and autoimmune diseases, including Crohn’s disease, celiac disease, Behcet’s disease, type 1 diabetes, and autoimmune neutropenia of early childhood (Franke et al., 2010; McGovern et al., 2010; Miyoshi et al., 2011; Rausch et al., 2011; Smyth et al., 2011; Ellinghaus et al., 2012; Forni et al., 2014; Tang et al., 2014; Maroni et al., 2015; Xavier et al., 2015; Ihara et al., 2017; Kløve-Mogensen et al., 2022). Overall, the null *FUT2* alleles appear to associate with an increased risk for reasons that are not yet understood. Nonetheless, associations with the microbiota composition and *FUT2* polymorphisms have been reported (Rausch et al., 2011; Wacklin et al., 2011; Tong et al., 2014; Wacklin et al., 2014; Gampa et al., 2017; Kumbhare et al., 2017; Rodríguez-Díaz et al., 2017; Pan et al., 2021; Rühlemann et al., 2021; Lopera-Maya et al., 2022). Although these remain debated and not fully consistent across studies (Davenport et al., 2016; Turpin et al., 2018), there are strong indications that microbiota composition is partly dependent on gut mucosal glycan composition through either bacterial adhesion molecules or through bacterial use of fucosylated glycans as nutrients (Coyne et al., 2005; Watanabe et al., 2010; Kashyap et al., 2013; Pickard et al., 2014; Wu et al., 2021).

Regulatory T cells are essential to control immune responses and the development of inflammatory and autoimmune diseases. In mice, FoxP3 + Treg cells induced by microbiota species of the Clostridium clusters are the dominant Treg cell subset in the gut (Atarashi et al., 2011; Nutsch and Hsieh, 2012). In humans, a likely counterpart has been identified, which we named double positive CD8α (DP8α Tregs), based on their expression of low CD8α levels together with CD4. Indeed, although they lack Foxp3, DP8α Tregs share with mouse clostridia-induced Tregs both the master transcription factor RORγt and a T Cell Receptor (TCR) reactivity to a clostridium species, namely *Faecalibacterium prausnitzii* (Clostridium cluster IV), supporting the role of this or a related bacterium in their induction (Jotereau et al., 2022). Abundant in the colonic lamina propria, DP8α Treg cells are also present in blood where they can be identified by their Foxp3^−^/CD4^+^/CD8α^low^/CxCR6^+^/CCR6^+^ phenotype (Godefroy et al., 2018). *Faecalibacterium prausnitzii* is one of the most abundant gut-associated clostridium-cluster’s member in healthy individuals and its decrease is associated with pathologies such as Inflammatory Bowel Diseases (IBD; Miquel et al., 2013). Interestingly, we also reported the striking and specific decrease of DP8α Treg cells in IBD patients, as compared to healthy controls or infectious colitis (Sarrabayrouse et al., 2014; Godefroy et al., 2018).

Considering this prior knowledge, we hypothesized that a link may exist between the risk of viral gastroenteritis and the abundance of microbiota-induced Treg cells so that individuals most susceptible to the virus infection, those with a *FUT2* wild-type allele (secretors), would acquire higher frequencies of Tregs that may contribute to their lower susceptibility to inflammatory diseases in comparison with those with two null alleles (nonsecretors). In an effort to start testing this hypothesis, we looked for a potential relationship between the presence of anti-rotavirus antibodies, the *FUT2* gene polymorphism, *F. prausnitzii* abundance and the level of peripheral DP8α Tregs in healthy young adults. We observed that serum anti-rotavirus antibodies were associated with the wild-type *FUT2* allele, as expected from earlier studies, and that the levels of DP8α Treg cells also associated with the wild-type allele, albeit only in individuals with high anti-rotavirus levels.

## Population, materials and methods

### Study design, participants and collection of samples

Peripheral blood, saliva and stool samples were obtained from healthy young adults, from 18 to 30 years old. Volunteers were recruited following a medical interview to ascertain that they had no known diseases, no known history of allergies, that they were not under medication, had no drugs intake, no recent alcohol intake and were non-smokers. The GOMMS PRL12009 project was part of the biobank of Biofortis SAS and was designed to enroll 80 participants. Of these, full sample collection and complete data could only be obtained from 72 individuals. This biocollection is registered at the French Research Ministry (AC-2013-1792) and the PRL12009 project was approved by the French Ethic Committee (CPP Ouest IV). All volunteers were aware of the study protocol and fulfilled the informed consent form.

### Analysis of the *FUT2* genetic polymorphism

The *FUT2* genetic polymorphism was analyzed by a combination of genotyping and of phenotyping of *FUT2*-dependent histo-blood group antigens (HBGAs). The major single Nucleotide Polymorphisms (SNPs) in the *FUT2* gene were investigated as described previously (Marionneau et al., 2005). HBGAs phenotypes were determined from buccal swabs specimens by enzyme-linked immunosorbent assay (ELISA), as described earlier (Loureiro Tonini et al., 2020). Briefly, saliva samples were first boiled for 10 min and then used at a dilution of 1:1,000 in a 0.1 M carbonate/bicarbonate buffer (pH 9,6) to coat 96-well microtiter plates (Maxisorp Nunc-Immuno plates, Thermo Scientific, CA, United States). Primary anti-carbohydrate monoclonal antibodies anti-A (ABO1 9113D10, Diagast, Loos, France), anti-B (B49), anti-Le^a^ (7LE) and anti-Le^b^ (2-25LE; Thermo Scientific, CA, United States) diluted at 1:400 in 5% milk/PBS were incubated for 1 h at 37°C. The lectin biotin-conjugated UEA-1 (Ulex Europaeus Agglutinin I—Vector Laboratories, CA, United States) was additionally used to detect the H antigen. Peroxidase–conjugated secondary reagents were used (Vector Laboratories, CA, United States) and reactions were developed with a 3,3′,5,5′-Tetramethylbenzidine kit (BD OptEIA, BD Biosciences). The cutoff value was defined as a twofold increase in absorbance value compared to the mean of two negative control samples.

### Analysis of circulating anti-RVA antibodies

*Neutralizing antibodies (NAbs)*: The assay was performed as previously described (Barbé et al., 2018). Briefly, serum samples diluted from 1:20 to 1:320 in serum-free medium were pre-incubated with 2 × 10^3^ FFU of trypsin-activated human G1P[8] RVA strain Wa for 1.5 h at 37°C prior to inoculation of MA104 cells. Plates were then incubated at 37°C for 45–90 min. After allowing virus attachment, the inoculum was removed and serum-supplemented medium was added. The infection was left to proceed for 14–15 h. Infected cells in methanol-fixed cell monolayers were detected by staining using a goat polyclonal anti-RV serum (Bio-Rad Antibodies) and FITC-labeled rabbit anti-goat IgG (Fc) antibody (Bio-Rad Antibodies), both diluted at 1:400 in PBS containing 3% BSA. Cell nuclei were stained with DAPI. Plates reading was performed on an ArrayScan HCS (ThermoScientific). The presence of neutralizing antibodies was considered when an inhibition > 50% in comparison with controls (absence of serum preincubation) was detected.

*Anti-rotavirus serum IgA*: Nunc Maxisorp Immunoplates were coated with a sheep anti-human rotavirus (Bio-Rad) diluted 1/500 in carbonate buffer pH 9.5 overnight at 4°C. Following a blocking step with 5% BSA/PBS for 1 h30 min at 37°C, plates were incubated for 1 h45 min at 37°C with trypsin-treated Human G1P[8] RVA strain Wa purified as previously described (Barbé et al., 2018) diluted in 1% BSA/PBS. Serum samples were then serially diluted from1/50 in 1% BSA/PBS and incubated for 1 h at 37°C. Then, detection of bound IgA was performed using a biotinylated anti-human IgA (Novus Biologicals) incubated for 1 h at 37°C, followed by peroxidase-conjugated streptavidin (Vector Labs) for 1 h at 37°C and reactions were developed with a 3,3′,5,5′-Tetramethylbenzidine kit (BD OptEIA, BD Biosciences). Between each step, plates were washed three times with 0.05% Tween/PBS. Titers were defined as the last dilution giving an OD_405_ value three times above the background obtained in absence of coating.

### Quantification of circulating DP8α Tregs

Peripheral blood mononuclear cells (PBMCs) were isolated by Ficoll gradient centrifugation. After isolation, PBMCs were stained for 45 min at 4°C in PBS 0.1% bovine serum albumin with the following antibodies: anti-human CD3-PE-Cy7 (clone UCHT1, BD Biosciences), anti-human CD4-FITC (clone 13B8.2, Beckman Coulter), anti-human CD8α-BV421 (clone RPA-T8, BD), anti-human CCR6-PE (clone G034E3, Biolegend), and anti-human CXCR6-APC (clone K041E5, Biolegend). Fluorescence was measured on a BD LSR II flow cytometer (BD Biosciences) and analyzed using FlowJo or DIVA softwares. DP8α Tregs (CD3^+^CD4^+^CD8α^lo^CCR6^+^CXCR6^+^ cells) were then quantified among total CD3^+^ T cells, as described previously (Godefroy et al., 2018). The gating strategy is shown in Supplementary Figure S2.

### Quantification of *Faecalibacterium prausnitzii* and 16S RNA metabarcoding

*Microbiota analysis*: Whole stools were collected in a fecotainer and immediately stored at 4°C upon transmission to the local lab (in less than 48 h) where 2 g of fresh feces were aliquoted and frozen at −80°C for molecular biology analyses. Genomic DNA of gut microbiota was released by a double lysis step: mechanical in a FastPrep24, MPBiomedicals and chemical with the Maxwell^®^ 16 Tissue DNA Purification Kit (Promega Corporation, Madison, WI, United States). DNA extraction was performed from one aliquot of 200 mg of frozen fecal sample and total genomic DNA was collected in a final volume of 200 μl. Double-stranded DNA (dsDNA) concentrations were measured by fluorimetry using the Qubit^®^ 2.0 Fluorometer and the Qubit^®^ dsDNA broad range assay (Invitrogen by Life Technologies, Carlsbad, CA, United States).

Polymerase chain reaction amplification was performed using 16S universal primers 341F and 785R targeting the V3–V4 region of the bacterial 16S ribosomal genes (Klindworth et al., 2013). The 16S V3–V4 amplicon size was verified by capillary electrophoresis (Agilent 2,100 Electrophoresis Bioanalyzer Instrument, Agilent technologies, Santa Clara, CA, United States). All amplicons were purified with magnetic beads using Agencourt AMPure XP beads (Beckman coulter, Brea, CA, United States). Then, for each sample, a sequencing library was generated by addition of dual indices and Illumina sequencing adapters, using a Nextera XT Index kit (Illumina, San Diego, CA, United States). Each library was cleaned with magnetic beads and its size was determined by capillary electrophoresis. After quantification by fluorimetry (Qubit^®^ 2.0 Fluorometer), libraries were normalized and pooled. The pool of libraries was further denatured and sequenced on the Illumina MiSeq platform, using a 2 × 250 paired-end Miseq kit V2 (Illumina, San Diego, CA, United States). Read sequences from fecal microbiota were analyzed using an in-house bioinformatic pipeline based on mothur v1.33.3 software (Schloss et al., 2009). Briefly, sequences were trimmed and aligned to the V3–V4 region of the 16S gene of the Greengenes database that had been formatted with mothur (gg_13_5_99 release). Chimera sequences were removed using the UCHIME algorithm. Reads were classified using a naive Bayesian classifier against RDP database release 11 with a bootstrap cut-off of 60%. Sequences were then clustered into operational taxonomic units (OTUs) using furthest-neighbor clustering at a similarity threshold of 97%.

*qPCR analysis*: Quantifications of *F*. *prausnitzii* were performed by qPCR using SYBR Green PCR Master Mix (Applied Biosystems) in a StepOnePlus apparatus (Applied Biosystems). Each reaction was done in duplicate in a final volume of 20 μl with 0.2 μM of each primer and 5 μl of the appropriate dilution of DNA. *F*. *prausnitzii* was |quantified using specific primers: sense, 5′-CCATGAATTGCCTTCAAAACTGTT-3′, and antisense, 5′-GAGCCTCAGCGTCAGTTGGT-3′ (Sokol et al., 2008). Appropriate dilution of stool DNA was assessed by performing an Internal Positive Control (AppliedBiosystems, Ref 4308323).

Amplifications were performed with the following temperature steps: 1 cycle at 95°C for 10 min. to denature DNA and activate polymerase, followed by 40 cycles of 95°C for 30 s., 60°C for 1 min. A dissociation step was added to control amplification specificity.

### Statistical analysis

GraphPad Prism v 9.0 (GraphPad Software, San Diego, CA, United States) was used for data analysis. Frequency distributions were analyzed by either Chi^2^ for trend or Fisher’s exact test. Comparisons of individual values between groups were performed using the Mann–Whitney test for continuous variables. Differences were considered statistically significant when the level of two-tailed significance was *p* < 0.05.

## Results

### The *FUT2* genotype associates with anti-RVA antibodies

**Figure 1:**
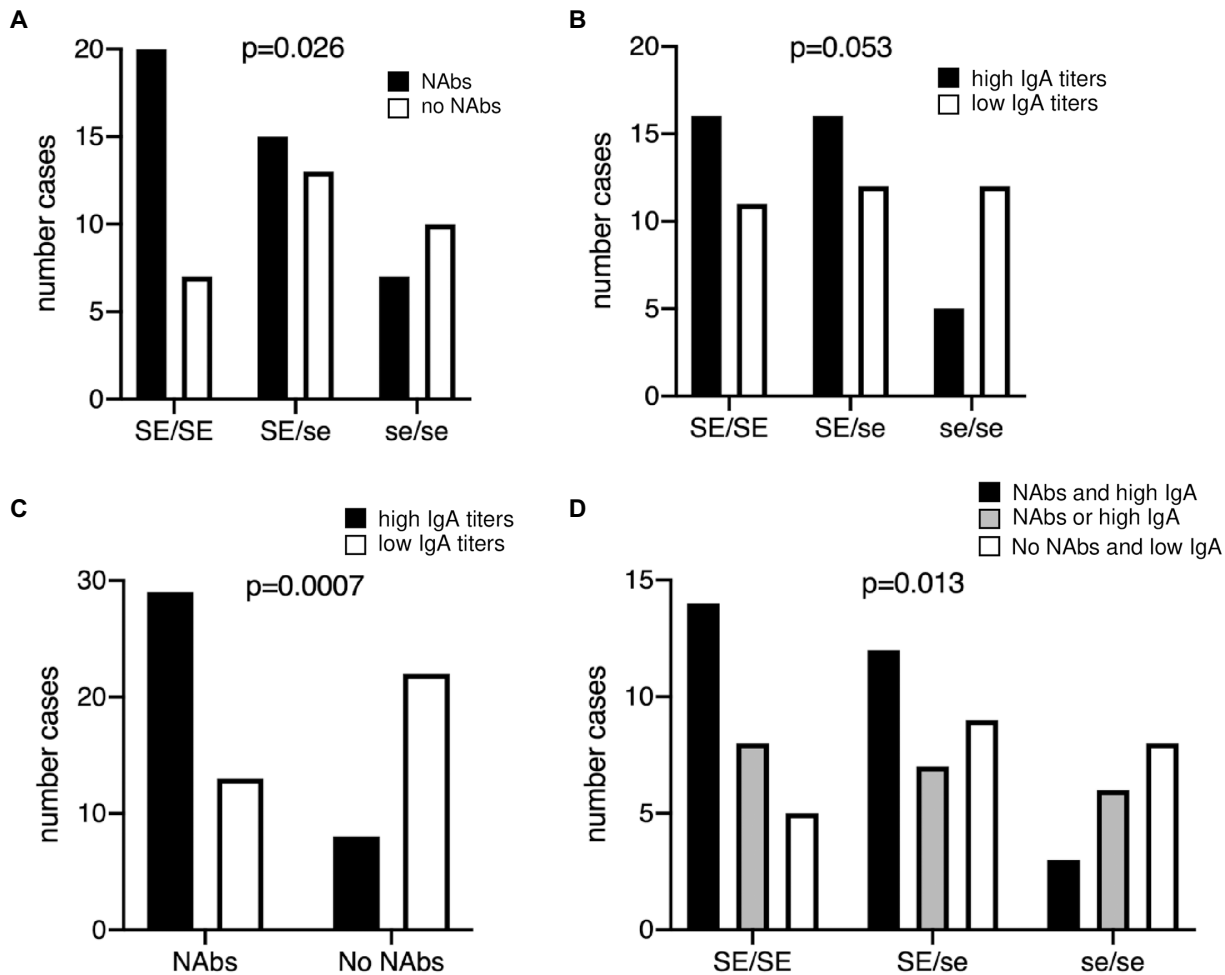
Relationship between the presence of anti-RVA antibodies and the *FUT2* genotype in healthy young adults. **(A)** Neutralizing antibodies **(**NAbs) titers against the RV strain Wa (G1P[8]) was determined, defining two groups of individuals according to the presence (black bars) or absence (white bars) of neutralizing antibodies, as depicted on Supplementary Figure S1. Chi-square test for trend was used to compare distributions according to the number of wild-type alleles (*p* = 0.026). **(B)** Serum anti-Wa IgA titers were classified as high (black bars) or low (white bars) as shown in Supplementary Figure S1 lower panel. Chi-square test for trend was used to compare distributions (*p* = 0.053). **(C)** Relationship between neutralizing antibodies and IgA titers against the Wa strain. Bars represent individuals with high (black) and low (white) IgA titers, respectively. Fisher’s exact test, *p* = 0.0007. **(D)** Individuals anti-RVA status was defined as strong in the presence of both NAbs and high IgA titers (black bars); intermediate either in absence of NAbs but high IgA or in presence of NAbs but low IgA (gray bars); weak in absence of NAbs and with low IgA titers (white bars). Chi-square test for trend was used to compare distributions (*p* = 0.013). SE/SE = *FUT2* homozygote wild-type; SE/se = *FUT2* heterozygotes; se/se = *FUT2* null homozygotes.

Since the presence of antibodies may reflect the history of infection by RVA, we sought to quantify anti-RVA serum antibodies in a cohort of healthy young French adults. Neutralizing antibodies (NAbs) are likely important, but do not appear to provide a correlate of protection (Desselberger, 2014; Caddy et al., 2020), we therefore additionally quantified serum IgA that constitutes a correlate of protection at the population level (Angel et al., 2012). The Wa strain was chosen as a target since it represents a dominant circulating genotype in western Europe (Desselberger, 2014) and since a vaccine based on an attenuated virus of the same genotype shows high efficacy in developed countries (Burnett et al., 2018). We observed that the distribution of NAbs was not homogeneous (Supplementary Figure S1, upper panel). It showed a group of individuals with NAbs, albeit at variable titers and a group lacking detectable NAbs. Therefore, in order to test a potential association with the *FUT2* genotype, serum samples were subdivided into two categories, those with or without NAbs, respectively. The distribution of anti-RVA IgAs appeared more normal all individuals, but one showing IgA responses with titers >1/50 (Supplementary Figure S1, lower panel). Samples were accordingly grouped into high and low titers based on their position above or below the median value, respectively. Analysis using the Fisher’s exact test indicated that the *FUT2* wild-type allele was associated with both the presence of neutralizing antibodies and high IgA titers, although the latter did not reach significance (Figures 1A,B). Some se/se individuals (nonsecretor phenotype) had either neutralizing antibodies or high IgA titers, indicating prior infection, which contrasted with earlier reports that showed a strong association with resistance to infection of nonsecretor children (Imbert-Marcille et al., 2014; Nordgren et al., 2014; Van Trang et al., 2014; Kambhampati et al., 2016; Zhang et al., 2016; Yang et al., 2017; Pérez-Ortín et al., 2019; Farahmand et al., 2021; Wang et al., 2021).

Infection episodes may not necessarily result in the generation of both NAbs and IgA. We therefore tested whether there existed an association between these two components of the anti-RVA immune response. As shown in Figure 1C, they were strongly associated. Nonetheless, a fair proportion of individuals presented divergent antibody profiles, having either high IgA titers but no NAbs or the opposite. Since a solid anti-RVA response likely comprises both neutralizing and IgA antibodies, we grouped individuals into three categories, namely those who have both NAbs and high IgA titers, those who have only one of these two components and those who have none of them. The distribution of these three categories of serum samples was then analyzed according to the *FUT2* genotype, which confirmed the association between the wild-type allele and a stronger anti-RVA immune status (Figure 1D). The difference in distribution of the three categories of serum samples within the three *FUT2* groups is striking. The wild-type homozygotes (SE/SE) presented an inverse distribution in comparison with mutant homozygotes (SE/SE; *p* < 0.05, Chi-square test), while heterozygotes (SE/se) showed an intermediate distribution. In addition, when considering phenotypes, that is comparing secretors (SE/SE + SE/se) versus nonsecretors (se/se), individuals who had both NAbs and high IgA titers were significantly over-represented among secretors in comparison with those who were classified as having either one or none of these two types of antibodies (*p* < 0.05, two-sided Fisher’s test). These data reveal that the wild-type *FUT2* allele (SE) is a risk factor for contracting RVA infections of sufficient magnitude to generate strong anti-viral immune responses (or inversely that null (se) alleles are protective).

### The *FUT2* genotype associates with DP8α Treg cells and *Faecalibacterium prausnitzii*

**Figure 2:**
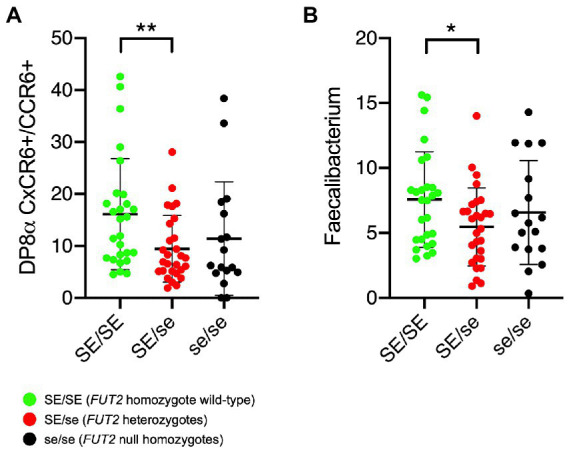
Relationship between the *FUT2* status and either DP8α Treg cells or the *Faecalibacterium prausnitzii* proportions. **(A)** Frequencies of circulating DP8α Treg cells quantified as described in the methods section (per 10.000 total CD3 + cells). **(B)** Relative abundance of the bacterium are given as percentage of the whole microbiota based on 16S metabarcoding. Comparisons were performed by two-sided Mann–Whitney test: ^*^p < 0.05; ^**^p < 0.01. SE/SE = *FUT2* homozygote wild-type (green symbols); SE/se = *FUT2* heterozygotes (red symbols); se/se = *FUT2* null homozygotes (black symbols).

Since the presence of *FUT2* null alleles correlate with the risk of several inflammatory or autoimmune diseases, and since *F. prausnitzii-*reactive DP8α Tregs appear to protect against intestinal inflammation (Touch et al., 2022), we tested whether the frequency of circulating DP8α Tregs or fecal *F. prausnitzii* abundance associated with the *FUT2* status. The gating strategy used to quantify DP8α Tregs is shown in Supplementary Figure S2. Within T cells, double positive CD4^+^/CD8^low^ expressing both CCR6 and CxCR6, which phenotype targets *F. prausnitzii*-reactive cells (Godefroy et al., 2018), were analyzed. Despite expected high individual variations, *FUT2* wild-type homozygous individuals appeared to present significantly higher frequencies of both DP8α Treg cells and their TCR-specific bacteria species. However, no difference between heterozygotes (SE/se) and null homozygotes (se/se) was apparent (Figures 2A,B). Quantification of *F. prausnitzii* by PCR (absolute quantification) rather than by 16S RNA analysis (relative quantification) yielded the same result (Supplementary Figure S3).

**Figure 3:**
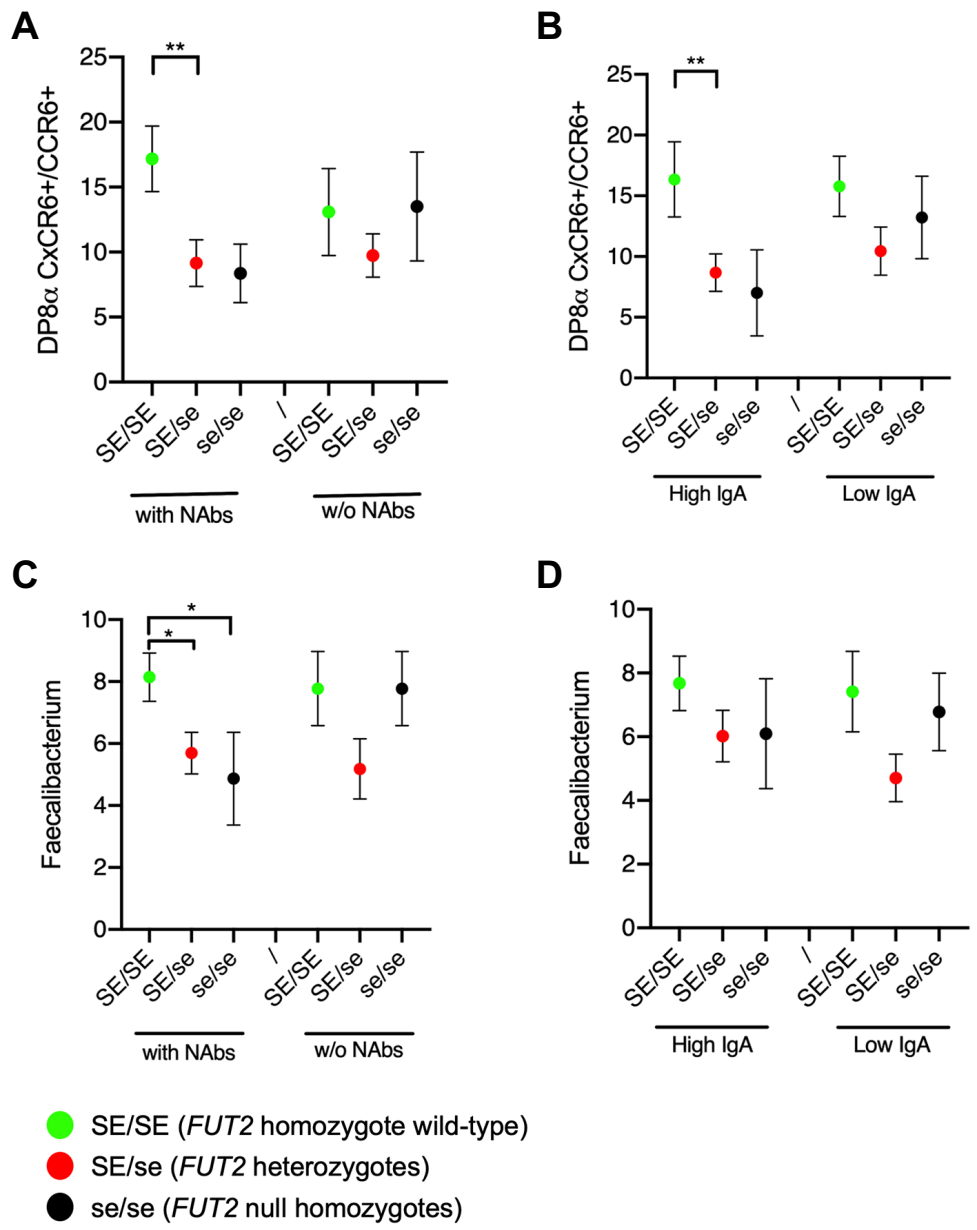
Tripartite relationships between the FUT2 status, the anti-RVA status and either the Treg cells or the *Faecalibacterium prausnitzii* frequency. **(A)** Frequencies of circulating DP8α Treg cells (per 10.000 total CD3+ cells) according to the *FUT2* status and the presence or absence of NAbs; **(B)** or the high versus low IgA titers; **(C)** Relative abundance of *Faecalibacterium* based on 16S metabarcoding according to the *FUT2* status and the presence or absence of NAbs; **(D)** or the high versus low IgA titers. Comparisons were performed by two-sided Mann–Whitney test: ^*^p < 0.05; ^**^p < 0.01. SE/SE = *FUT2* homozygote wild-type (green symbols); SE/se = *FUT2* heterozygotes (red symbols); se/se = *FUT2* null homozygotes (black symbols).

We next sought to determine whether the above-described association was influenced by the anti-RVA immune status. To this aim, the analyses shown in Figure 2 were replicated after splitting groups of individuals of each genotype according to either the presence of NAbs or the IgA titers. It appeared that the association between the frequency of DP8α Tregs and *FUT2* wild-type allele homozygosity was visible only among volunteers who had either NAbs (Figure 3A) or high titered anti-RVA IgAs (Figure 3B). Similar observations, albeit less clear-cut, were made for the relative abundance of *F. prausnitzii* that tended to be higher among serum samples from individuals with the *FUT2* wild-type allele who had either NAbs (Figure 3C) or high anti-RVA IgAs (Figure 3D).

## Discussion

Previous studies indicated that the risk for P[8] RVA gastroenteritis of sufficient severity to lead to hospital visit was strongly associated with the wild-type *FUT2* allele (Monedero et al., 2018; Ramani and Giri, 2019; Le Pendu and Ruvoën-Clouet, 2020; Sharma et al., 2020; Faden and Schaefer, 2021). We observed here that nonsecretors (se/se) had anti-RVA antibodies and often showed neutralizing antibodies, indicating that they can be infected. This is consistent with the observation that antibody responses to live vaccines containing a P[8] coding gene are present, but lower in nonsecretor children in comparison with secretor children (Kazi et al., 2017; Bucardo et al., 2018; Lee et al., 2018; Armah et al., 2019; Magwira et al., 2020). The reported near complete absence of nonsecretor children among those visiting at the hospital indicates that these children do get infected, but remain either asymptomatic or have mild disease only.

*FUT2* null alleles and the nonsecretor phenotype have been consistently associated with autoimmune and inflammatory diseases through many studies. Since dysbiosis is a hallmark of inflammatory diseases, it has been hypothesized that the *FUT2* gene would contribute to microbiota composition (Xavier et al., 2015; Imhann et al., 2018; Giampaoli et al., 2020; Zhou et al., 2020). Fucosylation would support a protective microbiota, whereas lack of fucosylation would allow the overgrowth of inflammatory bacteria. Several reports showed associations between gut microbiota composition and *FUT2* polymorphisms. However, it appeared that initial studies were underpowered since later studies that included larger number of individuals failed to reproduce the effect and since bacterial species reportedly increased or decreased according to the expressed *FUT2* alleles were not consistent across studies (Davenport et al., 2016; Turpin et al., 2018). It does not mean that these associations do not exist, but they may be obscured by the large diversity of the human gut microbiota and by the possibility that *FUT2* or HBGAs influences take place at the strain level rather than at the genus or species levels. Thus, a human gut symbiont, *Ruminococcus gravis* was recently reported to display a strain-specific repertoire of glycosidases, one of them showing specificity for a blood group A tetrasaccharide (Wu et al., 2021). Microbiota composition could also be affected by the strain-specific display of HBGA-specific bacterial adhesins such as the blood group A and B-specific adhesin of a *Lactobacillus mucosae* strain (Watanabe et al., 2010). Recent studies including over 7,000 participants found strong associations at the taxa and metabolic pathways levels with the ABO blood group. The associations were relying on the secretor phenotype (Rühlemann et al., 2021; Lopera-Maya et al., 2022). Further, animal studies showed convincing associations with HBGAs. A recent study in pigs reported a strong association between blood group A expression and gut microbiota composition (Yang et al., 2022). Likewise, the microbiota composition of *Fut2* KO mice diverges from that of their wild-type littermates and functionally increases susceptibility to induced gut inflammation, fucosylation modulating interactions with nonpathogenic resident microbes (Hooper and Gordon, 2001; Rausch et al., 2011; Kashyap et al., 2013; Garber et al., 2021).

Here, we further observed that the wild-type *FUT2* allele was associated with higher proportions of circulating DP8α Treg cells and of their target bacterium *F. prausnitzii*, albeit only among individuals who presented either neutralizing antibodies or high levels of IgA antibodies, linking the anti-RVA immune response to the level of microbiota-induced Treg cells. The association was detected among homozygote secretors (SE/SE), whilst heterozygotes (SE/se) presented similar levels of these Treg cells as nonsecretors (se/se), suggesting a difference between homozygote and heterozygote secretor individuals. Indeed a higher level of the fucosylated H type 1 epitope in SE/SE saliva samples in comparison with SE/se samples was previously reported, indicating a dose effect of the wild-type allele on fucosylation (Marionneau et al., 2005). Furthermore, we observed here that the levels of anti-rotavirus antibodies in SE/se serum samples were intermediate between those of the homozygous SE/SE and se/se (Figure 1D), suggesting that the strength of the anti-rotavirus immune response is influenced by the *FUT2* genotype and not only by the secretor phenotype. Homozygote secretors (SE/SE) might have an initial higher viral load than heterozygotes, and/or develop a more severe disease upon infection. Further studies are required to clarify this issue. In any case, it appears that infection that generates a strong anti-RVA immune response, likely because of a rather severe gastroenteritis, is associated with the presence of higher levels of microbiota-induced Treg cells. Interestingly, our team recently showed that these human Treg cells stimulate IgA synthesis *in vitro* (Jotereau et al., 2022), suggesting that they might represent an important player of the anti-viral response in case of infection by enteric viruses. Moreover, supporting a role for DP8α Tregs in intestinal homeostasis, low levels of these cells are associated with IBD (Godefroy et al., 2018), and we documented their ability to protect against intestinal inflammation in murine models (Touch et al., 2022; and unpublished results). Similarly, fecal *F. prausnitzii* levels are diminished in patients with IBD, as compared to healthy individuals (Sokol et al., 2006, 2009) and the anti-inflammatory effect of the bacterium has been well documented (Sokol et al., 2008; Martín et al., 2014; Zhang et al., 2014; Quévrain et al., 2016). Based on these observations, we hypothesized that the immune response against symptomatic RVA infection in early childhood would involve the development/expansion of microbiota-induced Treg cells that could be maintained in adulthood, thereby contributing to a balanced immunological state protective against both enteric viral infection and inflammatory diseases. How modulation of Treg cells levels is induced during the course of the anti-viral immune response remains to be determined, but it likely involves microbiota species abundance since their generation requires presentation of specific bacteria-derived epitopes by dendritic cells (Alameddine et al., 2019).

The limited number of volunteers enrolled in the present study did not allow to analyze an additional potential contribution of the ABO and Lewis polymorphisms (*ABO* and *FUT3* genes), since there were too few individuals in the resulting subgroups for meaningful comparisons. Studies involving larger cohorts of healthy adults would be required for confirmation and to study the effect of the combined *FUT2*, *ABO* and *FUT3* polymorphisms. Also, it would be interesting to analyze whether norovirus infection associates similarly with the frequency of Treg cells. An additional issue is that the present observations were obtained from young adults whilst RVA gastroenteritis is mainly occurring before 5 years of age. It is therefore unclear if the detected antibodies reflect early childhood infections or more recent asymptomatic reinfections. Studies on children with acute gastroenteritis and autoimmune diseases are thus warranted.

If our hypothesis nonetheless proved true, that is if immune response against symptomatic RVA infection in early childhood involves the development/expansion of microbiota-induced Treg cells, it would be important to ask whether RVA vaccines expand these Tregs, akin to symptomatic infections. If that was not the case, vaccinated children being protected from symptomatic gastroenteritis would fail to sufficiently expand microbiota-induced Treg cells and therefore might be at a higher risk of developing inflammatory and autoimmune diseases in adulthood. Comparing the levels of microbiota-induced Treg cells between vaccinated and nonvaccinated children or young adults would be a first step to explore this possible long-term side effect of rotavirus vaccines.

In conclusion, we observed an association between the *FUT2* genotype, anti-RVA antibodies and microbiota-induced Tregs frequency, suggesting that symptomatic RVA infection leads to the development of these Treg cells that will later contribute to immune homeostasis. It is interesting to observe that genetic polymorphisms selected in the context of a host-pathogen co-evolution involving young children might affect susceptibility to inflammation much later in life. This could have important implications for the development of future vaccines, warranting further studies.

## Data availability statement

The data presented in the study are deposited at the European Nucleotide Archive repository, accession number ERP144000: https://www.ebi.ac.uk/ena/browser/view/PRJEB58917.

## Ethics statement

The studies involving human participants were reviewed and approved by French Ethic Committee (CPP Ouest IV). The patients/participants provided their written informed consent to participate in this study.

## Author contributions

JLP conceived and designed the study and wrote the manuscript. JLP, NR-C, and FJ supervised the study. EG, LB, BM-V, JR, AB, TC, SL, DM, and J-MC carried out the experiments and discussed results. EG, TC, and JLP analyzed the data. All authors contributed to the article and approved the submitted version.

## Funding

This work was supported by an ANR-DGOS grant, program CE17, grant #15-0007-01 and by a Mérieux Research Grant, GOMMs, to JLP.

## Conflict of interest

SL and TC are employed by Biofortis Merieux Nutrisciences.

The remaining authors declare that the research was conducted in absence of any commercial or financial relationship that could be construed as a potential conflict of interest.

## Publisher’s note

All claims expressed in this article are solely those of the authors and do not necessarily represent those of their affiliated organizations, or those of the publisher, the editors and the reviewers. Any product that may be evaluated in this article, or claim that may be made by its manufacturer, is not guaranteed or endorsed by the publisher.

## References

[ref1] Alameddine J. Godefroy E. Papargyris L. Sarrabayrouse G. Tabiasco J. Bridonneau C.. (2019). Faecalibacterium prausnitzii skews human DC to prime IL10-producing T cells through TLR2/6/JNK signaling and IL-10, IL-27, CD39, and IDO-1 induction. Front. Immunol. 10:143. doi: 10.3389/fimmu.2019.00143, PMID: 30787928 PMC6373781

[ref2] Angel J. Franco M. A. Greenberg H. B. (2012). Rotavirus immune responses and correlates of protection. Curr. Opin. Virol. 2, 419–425. doi: 10.1016/j.coviro.2012.05.003, PMID: 22677178 PMC3422408

[ref3] Armah G. E. Cortese M. M. Dennis F. E. Yu Y. Morrow A. L. McNeal M. M.. (2019). Rotavirus vaccine take in infants is associated with secretor status. J. Infect. Dis. 219, 746–749. doi: 10.1093/infdis/jiy573, PMID: 30357332

[ref4] Atarashi K. Tanoue T. Shima T. Imaoka A. Kuwahara T. Momose Y.. (2011). Induction of colonic regulatory T cells by indigenous *Clostridium* species. Science 331, 337–341. doi: 10.1126/science.1198469, PMID: 21205640 PMC3969237

[ref5] Bányai K. Estes M. K. Martella V. Parashar U. D. (2018). Viral gastroenteritis. Lancet 392, 175–186. doi: 10.1016/S0140-6736(18)31128-0, PMID: 30025810 PMC8883799

[ref6] Barbé L. Le Moullac-Vaidye B. Echasserieau K. Bernardeau K. Carton T. Bovin N.. (2018). Histo-blood group antigen-binding specificities of human rotaviruses are associated with gastroenteritis but not with in vitro infection. Sci. Rep. 8:12961. doi: 10.1038/s41598-018-31005-4, PMID: 30154494 PMC6113245

[ref7] Bucardo F. Nordgren J. Reyes Y. Gonzalez F. Sharma S. Svensson L. (2018). The Lewis a phenotype is a restriction factor for Rotateq and Rotarix vaccine-take in Nicaraguan children. Sci. Rep. 8:1502. doi: 10.1038/s41598-018-19718-y, PMID: 29367698 PMC5784145

[ref8] Burnett E. Parashar U. Tate J. (2018). Rotavirus vaccines: effectiveness, safety, and future directions. Paediatr. Drugs 20, 223–233. doi: 10.1007/s40272-018-0283-3, PMID: 29388076 PMC5955791

[ref9] Caddy S. L. Vaysburd M. Wing M. Foss S. Andersen J. T. O’Connell K.. (2020). Intracellular neutralisation of rotavirus by VP6-specific IgG. PLoS Pathog. 16:e1008732. doi: 10.1371/journal.ppat.1008732, PMID: 32750093 PMC7428215

[ref10] Cooling L. (2015). Blood groups in infection and host susceptibility. Clin. Microbiol. Rev. 28, 801–870. doi: 10.1128/CMR.00109-14, PMID: 26085552 PMC4475644

[ref11] Coyne M. J. Reinap B. Lee M. M. Comstock L. E. (2005). Human symbionts use a host-like pathway for surface fucosylation. Science 307, 1778–1781. doi: 10.1126/science.1106469, PMID: 15774760

[ref12] Davenport E. R. Goodrich J. K. Bell J. T. Spector T. D. Ley R. E. Clark A. G. (2016). ABO antigen and secretor statuses are not associated with gut microbiota composition in 1,500 twins. BMC Genomics 17:941. doi: 10.1186/s12864-016-3290-1, PMID: 27871240 PMC5117602

[ref13] Desselberger U. (2014). Rotaviruses. Virus Res. 190, 75–96. doi: 10.1016/j.virusres.2014.06.016 25016036

[ref14] Ellinghaus D. Ellinghaus E. Nair R. P. Stuart P. E. Esko T. Metspalu A.. (2012). Combined analysis of genome-wide association studies for Crohn disease and psoriasis identifies seven shared susceptibility loci. Am. J. Hum. Genet. 90, 636–647. doi: 10.1016/j.ajhg.2012.02.020, PMID: 22482804 PMC3322238

[ref15] Faden H. Schaefer B. A. (2021). Secretors of HBGA and susceptibility to norovirus and rotavirus diarrhea. Pediatr. Infect. Dis. J. 40, 846–851. doi: 10.1097/INF.0000000000003218, PMID: 34397778

[ref16] Farahmand M. Jalilvand S. Arashkia A. Shahmahmoodi S. Afchangi A. Mollaei-Kandelous Y.. (2021). Association between circulating rotavirus genotypes and histo-blood group antigens in the children hospitalized with acute gastroenteritis in Iran. J. Med. Virol. 93, 4817–4823. doi: 10.1002/jmv.26808, PMID: 33463743

[ref17] Ferrer-Admetlla A. Sikora M. Laayouni H. Esteve A. Roubinet F. Blancher A.. (2009). A natural history of FUT2 polymorphism in humans. Mol. Biol. Evol. 26, 1993–2003. doi: 10.1093/molbev/msp108, PMID: 19487333

[ref18] Forni D. Cleynen I. Ferrante M. Cassinotti A. Cagliani R. Ardizzone S.. (2014). ABO histo-blood group might modulate predisposition to Crohn’s disease and affect disease behavior. J. Crohns Colitis 8, 489–494. doi: 10.1016/j.crohns.2013.10.014, PMID: 24268527

[ref19] Franke A. McGovern D. P. B. Barrett J. C. Wang K. Radford-Smith G. L. Ahmad T.. (2010). Genome-wide meta-analysis increases to 71 the number of confirmed Crohn’s disease susceptibility loci. Nat. Genet. 42, 1118–1125. doi: 10.1038/ng.717, PMID: 21102463 PMC3299551

[ref20] Gampa A. Engen P. A. Shobar R. Mutlu E. A. (2017). Relationships between gastrointestinal microbiota and blood group antigens. Physiol. Genomics 49, 473–483. doi: 10.1152/physiolgenomics.00043.2017, PMID: 28710295 PMC5625272

[ref21] Garber J. M. Hennet T. Szymanski C. M. (2021). Significance of fucose in intestinal health and disease. Mol. Microbiol. 115, 1086–1093. doi: 10.1111/mmi.14681, PMID: 33434389

[ref22] Giampaoli O. Conta G. Calvani R. Miccheli A. (2020). Can the FUT2 non-secretor phenotype associated with gut microbiota increase the children susceptibility for type 1 diabetes? A Mini Review. Front. Nutr. 7:606171. doi: 10.3389/fnut.2020.606171, PMID: 33425974 PMC7785815

[ref23] Godefroy E. Alameddine J. Montassier E. Mathé J. Desfrançois-Noël J. Marec N.. (2018). Expression of CCR6 and CXCR6 by gut-derived CD4+/CD8α+ T-regulatory cells, which are decreased in blood samples from patients with inflammatory bowel diseases. Gastroenterology 155, 1205–1217. doi: 10.1053/j.gastro.2018.06.078, PMID: 29981781

[ref24] Hooper L. V. Gordon J. I. (2001). Glycans as legislators of host–microbial interactions: spanning the spectrum from symbiosis to pathogenicity. Glycobiology 11, 1R–10R. doi: 10.1093/glycob/11.2.1r, PMID: 11287395

[ref25] Ihara K. Fukano C. Ayabe T. Fukami M. Ogata T. Kawamura T.. (2017). FUT2 non-secretor status is associated with type 1 diabetes susceptibility in Japanese children. Diabet. Med. 34, 586–589. doi: 10.1111/dme.13288, PMID: 27859559

[ref26] Imbert-Marcille B.-M. Barbé L. Dupé M. Le Moullac-Vaidye B. Besse B. Peltier C.. (2014). A FUT2 gene common polymorphism determines resistance to rotavirus a of the P[8] genotype. J. Infect. Dis. 209, 1227–1230. doi: 10.1093/infdis/jit655, PMID: 24277741

[ref27] Imhann F. Vich Vila A. Bonder M. J. Fu J. Gevers D. Visschedijk M. C.. (2018). Interplay of host genetics and gut microbiota underlying the onset and clinical presentation of inflammatory bowel disease. Gut 67, 108–119. doi: 10.1136/gutjnl-2016-312135, PMID: 27802154 PMC5699972

[ref28] Jotereau F. Alameddine J. Teusan R. Pédron A. Jouand N. Altare F.. (2022). Human gut microbiota-reactive DP8α regulatory T cells, signature and related emerging functions. Front. Immunol. 13:1026994. doi: 10.3389/fimmu.2022.1026994, PMID: 36479125 PMC9720269

[ref29] Kambhampati A. Payne D. C. Costantini V. Lopman B. A. (2016). Host genetic susceptibility to enteric viruses: a systematic review and Metaanalysis. Clin. Infect. Dis. 62, 11–18. doi: 10.1093/cid/civ873, PMID: 26508510 PMC4679673

[ref30] Kashyap P. C. Marcobal A. Ursell L. K. Smits S. A. Sonnenburg E. D. Costello E. K.. (2013). Genetically dictated change in host mucus carbohydrate landscape exerts a diet-dependent effect on the gut microbiota. Proc. Natl. Acad. Sci. U. S. A. 110, 17059–17064. doi: 10.1073/pnas.1306070110, PMID: 24062455 PMC3800993

[ref31] Kazi A. M. Cortese M. M. Yu Y. Lopman B. Morrow A. L. Fleming J. A.. (2017). Secretor and salivary ABO blood group antigen status predict rotavirus vaccine take in infants. J. Infect. Dis. 215, 786–789. doi: 10.1093/infdis/jix028, PMID: 28329092

[ref32] Klindworth A. Pruesse E. Schweer T. Peplies J. Quast C. Horn M.. (2013). Evaluation of general 16S ribosomal RNA gene PCR primers for classical and next-generation sequencing-based diversity studies. Nucleic Acids Res. 41:e1. doi: 10.1093/nar/gks808, PMID: 22933715 PMC3592464

[ref33] Kløve-Mogensen K. Steffensen R. Masmas T. N. Glenthøj A. Haunstrup T. M. Ratcliffe P.. (2022). ABO, secretor, and Lewis carbohydrate histo-blood groups are associated with autoimmune neutropenia of early childhood in Danish patients. Transfusion 62, 1636–1642. doi: 10.1111/trf.17002, PMID: 35792132 PMC9544446

[ref34] Kumbhare S. V. Kumar H. Chowdhury S. P. Dhotre D. P. Endo A. Mättö J.. (2017). A cross-sectional comparative study of gut bacterial community of Indian and Finnish children. Sci. Rep. 7:10555. doi: 10.1038/s41598-017-11215-y, PMID: 28874767 PMC5585376

[ref35] Le Pendu J. Nyström K. Ruvoën-Clouet N. (2014). Host-pathogen co-evolution and glycan interactions. Curr. Opin. Virol. 7, 88–94. doi: 10.1016/j.coviro.2014.06.001, PMID: 25000207

[ref36] Le Pendu J. Ruvoën-Clouet N. (2020). Fondness for sugars of enteric viruses confronts them with human glycans genetic diversity. Hum. Genet. 139, 903–910. doi: 10.1007/s00439-019-02090-w, PMID: 31760489

[ref37] Lee B. Dickson D. M. deCamp A. C. Ross Colgate E. Diehl S. A. Uddin M. I.. (2018). Histo-blood group antigen phenotype determines susceptibility to genotype-specific rotavirus infections and impacts measures of rotavirus vaccine efficacy. J. Infect. Dis. 217, 1399–1407. doi: 10.1093/infdis/jiy054, PMID: 29390150 PMC5894073

[ref38] Lopera-Maya E. A. Kurilshikov A. van der Graaf A. Hu S. Andreu-Sánchez S. Chen L.. (2022). Effect of host genetics on the gut microbiome in 7,738 participants of the Dutch microbiome project. Nat. Genet. 54, 143–151. doi: 10.1038/s41588-021-00992-y, PMID: 35115690

[ref39] Loureiro Tonini M. A. Pires Gonçalves Barreira D. M. Bueno de Freitas Santolin L. Bondi Volpini L. P. Gagliardi Leite J. P. Le Moullac-Vaidye B.. (2020). FUT2, secretor status and FUT3 polymorphisms of children with acute diarrhea infected with rotavirus and norovirus in Brazil. Viruses 12:E1084. doi: 10.3390/v12101084, PMID: 32992989 PMC7600990

[ref40] Magwira C. A. Kgosana L. P. Esona M. D. Seheri M. L. (2020). Low fecal rotavirus vaccine virus shedding is significantly associated with non-secretor histo-blood group antigen phenotype among infants in northern Pretoria, South Africa. Vaccine 38, 8260–8263. doi: 10.1016/j.vaccine.2020.11.025, PMID: 33213928

[ref41] Marionneau S. Airaud F. Bovin N. V. Le Pendu J. Ruvoën-Clouet N. (2005). Influence of the combined ABO, FUT2, and FUT3 polymorphism on susceptibility to Norwalk virus attachment. J. Infect. Dis. 192, 1071–1077. doi: 10.1086/432546, PMID: 16107962

[ref42] Marionneau S. Cailleau-Thomas A. Rocher J. Le Moullac-Vaidye B. Ruvoën-clouet N. Clément M.. (2001). ABH and Lewis histo-blood group antigens, a model for the meaning of oligosaccharide diversity in the face of a changing world. Biochimie 83, 565–573. doi: 10.1016/S0300-9084(01)01321-9, PMID: 11522384

[ref43] Maroni L. van de Graaf S. F. J. Hohenester S. D. Oude Elferink R. P. J. Beuers U. (2015). Fucosyltransferase 2: a genetic risk factor for primary sclerosing cholangitis and Crohn’s disease--a comprehensive review. Clin. Rev. Allergy Immunol. 48, 182–191. doi: 10.1007/s12016-014-8423-1 24828903

[ref44] Martín R. Chain F. Miquel S. Lu J. Gratadoux J.-J. Sokol H.. (2014). The commensal bacterium *Faecalibacterium prausnitzii* is protective in DNBS-induced chronic moderate and severe colitis models. Inflamm. Bowel Dis. 20, 417–430. doi: 10.1097/01.MIB.0000440815.76627.64, PMID: 24418903

[ref45] McGovern D. P. B. Jones M. R. Taylor K. D. Marciante K. Yan X. Dubinsky M.. (2010). Fucosyltransferase 2 (FUT2) non-secretor status is associated with Crohn’s disease. Hum. Mol. Genet. 19, 3468–3476. doi: 10.1093/hmg/ddq248, PMID: 20570966 PMC2916706

[ref46] Miquel S. Martín R. Rossi O. Bermúdez-Humarán L. G. Chatel J. M. Sokol H.. (2013). *Faecalibacterium prausnitzii* and human intestinal health. Curr. Opin. Microbiol. 16, 255–261. doi: 10.1016/j.mib.2013.06.003 23831042

[ref47] Miyoshi J. Yajima T. Okamoto S. Matsuoka K. Inoue N. Hisamatsu T.. (2011). Ectopic expression of blood type antigens in inflamed mucosa with higher incidence of FUT2 secretor status in colonic Crohn’s disease. J. Gastroenterol. 46, 1056–1063. doi: 10.1007/s00535-011-0425-7, PMID: 21725903

[ref48] Monedero V. Buesa J. Rodríguez-Díaz J. (2018). The interactions between host Glycobiology, bacterial microbiota, and viruses in the gut. Viruses 10:96. doi: 10.3390/v10020096, PMID: 29495275 PMC5850403

[ref49] Nordgren J. Sharma S. Bucardo F. Nasir W. Günaydın G. Ouermi D.. (2014). Both Lewis and secretor status mediate susceptibility to rotavirus infections in a rotavirus genotype-dependent manner. Clin. Infect. Dis. 59, 1567–1573. doi: 10.1093/cid/ciu633, PMID: 25097083 PMC4650770

[ref50] Nordgren J. Svensson L. (2019). Genetic susceptibility to human norovirus infection: an update. Viruses 11:E226. doi: 10.3390/v11030226, PMID: 30845670 PMC6466115

[ref51] Nutsch K. M. Hsieh C.-S. (2012). T cell tolerance and immunity to commensal bacteria. Curr. Opin. Immunol. 24, 385–391. doi: 10.1016/j.coi.2012.04.009, PMID: 22613090 PMC3423487

[ref52] Pan C. Ning Y. Jia Y. Cheng S. Wen Y. Yang X.. (2021). Transcriptome-wide association study identified candidate genes associated with gut microbiota. Gut Pathog. 13:74. doi: 10.1186/s13099-021-00474-w, PMID: 34922623 PMC8684646

[ref53] Pérez-Ortín R. Vila-Vicent S. Carmona-Vicente N. Santiso-Bellón C. Rodríguez-Díaz J. Buesa J. (2019). Histo-blood group antigens in children with symptomatic rotavirus infection. Viruses 11:E339. doi: 10.3390/v11040339, PMID: 30974776 PMC6520971

[ref54] Pickard J. M. Maurice C. F. Kinnebrew M. A. Abt M. C. Schenten D. Golovkina T. V.. (2014). Rapid fucosylation of intestinal epithelium sustains host-commensal symbiosis in sickness. Nature 514, 638–641. doi: 10.1038/nature13823, PMID: 25274297 PMC4214913

[ref55] Quévrain E. Maubert M. A. Michon C. Chain F. Marquant R. Tailhades J.. (2016). Identification of an anti-inflammatory protein from *Faecalibacterium prausnitzii*, a commensal bacterium deficient in Crohn’s disease. Gut 65, 415–425. doi: 10.1136/gutjnl-2014-307649, PMID: 26045134 PMC5136800

[ref56] Ramani S. Giri S. (2019). Influence of histo blood group antigen expression on susceptibility to enteric viruses and vaccines. Curr. Opin. Infect. Dis. 32, 445–452. doi: 10.1097/QCO.0000000000000571, PMID: 31335438

[ref57] Ramani S. Hu L. Venkataram Prasad B. V. Estes M. K. (2016). Diversity in rotavirus-host glycan interactions: a “sweet” Spectrum. Cell. Mol. Gastroenterol. Hepatol. 2, 263–273. doi: 10.1016/j.jcmgh.2016.03.002, PMID: 28090561 PMC5042371

[ref58] Rausch P. Rehman A. Künzel S. Häsler R. Ott S. J. Schreiber S.. (2011). Colonic mucosa-associated microbiota is influenced by an interaction of Crohn disease and FUT2 (secretor) genotype. Proc. Natl. Acad. Sci. U. S. A. 108, 19030–19035. doi: 10.1073/pnas.1106408108, PMID: 22068912 PMC3223430

[ref59] Rodríguez-Díaz J. García-Mantrana I. Vila-Vicent S. Gozalbo-Rovira R. Buesa J. Monedero V.. (2017). Relevance of secretor status genotype and microbiota composition in susceptibility to rotavirus and norovirus infections in humans. Sci. Rep. 7:45559. doi: 10.1038/srep45559, PMID: 28358023 PMC5372083

[ref60] Rühlemann M. C. Hermes B. M. Bang C. Doms S. Moitinho-Silva L. Thingholm L. B.. (2021). Genome-wide association study in 8,956 German individuals identifies influence of ABO histo-blood groups on gut microbiome. Nat. Genet. 53, 147–155. doi: 10.1038/s41588-020-00747-1, PMID: 33462482

[ref61] Ruvoën-Clouet N. Belliot G. Le Pendu J. (2013). Noroviruses and histo-blood groups: the impact of common host genetic polymorphisms on virus transmission and evolution. Rev. Med. Virol. 23, 355–366. doi: 10.1002/rmv.1757, PMID: 23959967

[ref62] Sarrabayrouse G. Bossard C. Chauvin J.-M. Jarry A. Meurette G. Quévrain E.. (2014). CD4CD8αα lymphocytes, a novel human regulatory T cell subset induced by colonic bacteria and deficient in patients with inflammatory bowel disease. PLoS Biol. 12:e1001833. doi: 10.1371/journal.pbio.1001833, PMID: 24714093 PMC3979654

[ref63] Schloss P. D. Westcott S. L. Ryabin T. Hall J. R. Hartmann M. Hollister E. B.. (2009). Introducing mothur: open-source, platform-independent, community-supported software for describing and comparing microbial communities. Appl. Environ. Microbiol. 75, 7537–7541. doi: 10.1128/AEM.01541-09, PMID: 19801464 PMC2786419

[ref64] Schroten H. Hanisch F.-G. Hansman G. S. (2016). Human norovirus interactions with Histo-blood group antigens and human Milk oligosaccharides. J. Virol. 90, 5855–5859. doi: 10.1128/JVI.00317-16, PMID: 27122582 PMC4907220

[ref65] Sharma S. Hagbom M. Svensson L. Nordgren J. (2020). The impact of human genetic polymorphisms on rotavirus susceptibility, epidemiology, and vaccine take. Viruses 12:E324. doi: 10.3390/v12030324, PMID: 32192193 PMC7150750

[ref66] Silva L. M. Carvalho A. S. Guillon P. Seixas S. Azevedo M. Almeida R.. (2010). Infection-associated FUT2 (Fucosyltransferase 2) genetic variation and impact on functionality assessed by in vivo studies. Glycoconj. J. 27, 61–68. doi: 10.1007/s10719-009-9255-8, PMID: 19757028

[ref67] Smyth D. J. Cooper J. D. Howson J. M. M. Clarke P. Downes K. Mistry T.. (2011). FUT2 nonsecretor status links type 1 diabetes susceptibility and resistance to infection. Diabetes 60, 3081–3084. doi: 10.2337/db11-0638, PMID: 22025780 PMC3198057

[ref68] Sokol H. Pigneur B. Watterlot L. Lakhdari O. Bermúdez-Humarán L. G. Gratadoux J.-J.. (2008). *Faecalibacterium prausnitzii* is an anti-inflammatory commensal bacterium identified by gut microbiota analysis of Crohn disease patients. Proc. Natl. Acad. Sci. U. S. A. 105, 16731–16736. doi: 10.1073/pnas.0804812105, PMID: 18936492 PMC2575488

[ref69] Sokol H. Seksik P. Furet J. P. Firmesse O. Nion-Larmurier I. Beaugerie L.. (2009). Low counts of *Faecalibacterium prausnitzii* in colitis microbiota. Inflamm. Bowel Dis. 15, 1183–1189. doi: 10.1002/ibd.20903, PMID: 19235886

[ref70] Sokol H. Seksik P. Rigottier-Gois L. Lay C. Lepage P. Podglajen I.. (2006). Specificities of the fecal microbiota in inflammatory bowel disease. Inflamm. Bowel Dis. 12, 106–111. doi: 10.1097/01.MIB.0000200323.38139.c6 16432374

[ref71] Tan M. Jiang X. (2014). Histo-blood group antigens: a common niche for norovirus and rotavirus. Expert Rev. Mol. Med. 16:e5. doi: 10.1017/erm.2014.2, PMID: 24606759 PMC12406300

[ref72] Tang H. Jin X. Li Y. Jiang H. Tang X. Yang X.. (2014). A large-scale screen for coding variants predisposing to psoriasis. Nat. Genet. 46, 45–50. doi: 10.1038/ng.2827, PMID: 24212883

[ref73] Tenge V. R. Hu L. Prasad B. V. V. Larson G. Atmar R. L. Estes M. K.. (2021). Glycan recognition in human norovirus infections. Viruses 13:2066. doi: 10.3390/v13102066, PMID: 34696500 PMC8537403

[ref74] Tong M. McHardy I. Ruegger P. Goudarzi M. Kashyap P. C. Haritunians T.. (2014). Reprograming of gut microbiome energy metabolism by the FUT2 Crohn’s disease risk polymorphism. ISME J. 8, 2193–2206. doi: 10.1038/ismej.2014.64, PMID: 24781901 PMC4992076

[ref75] Touch S. Godefroy E. Rolhion N. Danne C. Oeuvray C. Straube M.. (2022). Human CD4+CD8α+ Tregs induced by *Faecalibacterium prausnitzii* protect against intestinal inflammation. JCI Insight 7:e154722. doi: 10.1172/jci.insight.154722, PMID: 35536673 PMC9309064

[ref76] Troeger C. Khalil I. A. Rao P. C. Cao S. Blacker B. F. Ahmed T.. (2018). Rotavirus vaccination and the global burden of rotavirus diarrhea among children younger than 5 years. JAMA Pediatr. 172, 958–965. doi: 10.1001/jamapediatrics.2018.1960, PMID: 30105384 PMC6233802

[ref77] Turpin W. Bedrani L. Espin-Garcia O. Xu W. Silverberg M. S. Smith M. I.. (2018). FUT2 genotype and secretory status are not associated with fecal microbial composition and inferred function in healthy subjects. Gut Microbes 9, 1–12. doi: 10.1080/19490976.2018.1445956, PMID: 29533703 PMC6219652

[ref78] Van Trang N. Vu H. T. Le N. T. Huang P. Jiang X. Anh D. D. (2014). Association between norovirus and rotavirus infection and histo-blood group antigen types in Vietnamese children. J. Clin. Microbiol. 52, 1366–1374. doi: 10.1128/JCM.02927-13, PMID: 24523471 PMC3993640

[ref79] Wacklin P. Mäkivuokko H. Alakulppi N. Nikkilä J. Tenkanen H. Räbinä J.. (2011). Secretor genotype (FUT2 gene) is strongly associated with the composition of Bifidobacteria in the human intestine. PLoS One 6:e20113. doi: 10.1371/journal.pone.0020113, PMID: 21625510 PMC3098274

[ref80] Wacklin P. Tuimala J. Nikkilä J. Tims S. Mäkivuokko H. Alakulppi N.. (2014). Faecal microbiota composition in adults is associated with the FUT2 gene determining the secretor status. PLoS One 9:e94863. doi: 10.1371/journal.pone.0094863, PMID: 24733310 PMC3986271

[ref81] Wang J.-X. Chen L.-N. Zhang C.-J. Zhou H.-L. Zhang Y.-H. Zhang X.-J.. (2021). Genetic susceptibility to rotavirus infection in Chinese children: a population-based case-control study. Hum. Vaccin. Immunother. 17, 1803–1810. doi: 10.1080/21645515.2020.1835121, PMID: 33295824 PMC8115749

[ref82] Watanabe M. Kinoshita H. Nitta M. Yukishita R. Kawai Y. Kimura K.. (2010). Identification of a new adhesin-like protein from lactobacillus mucosae ME-340 with specific affinity to the human blood group a and B antigens. J. Appl. Microbiol. 109, 927–935. doi: 10.1111/j.1365-2672.2010.04719.x, PMID: 20408914

[ref83] Wu H. Crost E. H. Owen C. D. van Bakel W. Martínez Gascueña A. Latousakis D.. (2021). The human gut symbiont Ruminococcus gnavus shows specificity to blood group a antigen during mucin glycan foraging: implication for niche colonisation in the gastrointestinal tract. PLoS Biol. 19:e3001498. doi: 10.1371/journal.pbio.3001498, PMID: 34936658 PMC8730463

[ref84] Xavier J. M. Shahram F. Sousa I. Davatchi F. Matos M. Abdollahi B. S.. (2015). FUT2: filling the gap between genes and environment in Behçet’s disease? Ann. Rheum. Dis. 74, 618–624. doi: 10.1136/annrheumdis-2013-204475, PMID: 24326010

[ref85] Yang T.-A. Hou J.-Y. Huang Y.-C. Chen C.-J. (2017). Genetic susceptibility to rotavirus gastroenteritis and vaccine effectiveness in Taiwanese children. Sci. Rep. 7:6412. doi: 10.1038/s41598-017-06686-y, PMID: 28743921 PMC5526899

[ref86] Yang H. Wu J. Huang X. Zhou Y. Zhang Y. Liu M.. (2022). ABO genotype alters the gut microbiota by regulating GalNAc levels in pigs. Nature 606, 358–367. doi: 10.1038/s41586-022-04769-z, PMID: 35477154 PMC9157047

[ref87] Zhang X.-F. Long Y. Tan M. Zhang T. Huang Q. Jiang X.. (2016). P[8] and P[4] rotavirus infection associated with secretor phenotypes among children in South China. Sci. Rep. 6:34591. doi: 10.1038/srep34591, PMID: 27708367 PMC5052604

[ref88] Zhang M. Qiu X. Zhang H. Yang X. Hong N. Yang Y.. (2014). Faecalibacterium prausnitzii inhibits interleukin-17 to ameliorate colorectal colitis in rats. PLoS One 9:e109146. doi: 10.1371/journal.pone.0109146, PMID: 25275569 PMC4183556

[ref89] Zhou R. Llorente C. Cao J. Gao B. Duan Y. Jiang L.. (2020). Deficiency of intestinal α1-2-Fucosylation exacerbates ethanol-induced liver disease in mice. Alcohol. Clin. Exp. Res. 44, 1842–1851. doi: 10.1111/acer.14405, PMID: 32628772 PMC7808344

