# Supplementary material for: Microbiota-induced regulatory T cells associate with FUT2-dependent susceptibility to rotavirus gastroenteritis
Source: Front Microbiol. 2023 Feb 27;14:1123803. doi: 10.3389/fmicb.2023.1123803 (PMC10008897; doi:10.3389/fmicb.2023.1123803)
Supplement: Supplementary file 1 [file Data_Sheet_1.docx]

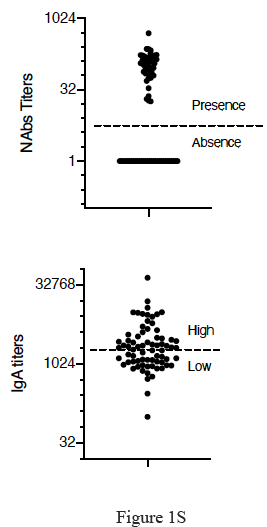


**Figure 1S:** Distribution of the anti-Wa strain neutralizing antibodies and IgA titers in the serum samples of 72 young French adult volunteers. Samples were grouped for analysis according to either the presence or absence of neutralizing antibodies (upper panel) or high (above median) and low IgA titers (below median) (lower panel). Neutralizing antibodies titers correspond to the highest serum dilution that decreases infected-cells number >50% of controls. IgA titers correspond to the last serum dilution that gave OD values 3 times above background.


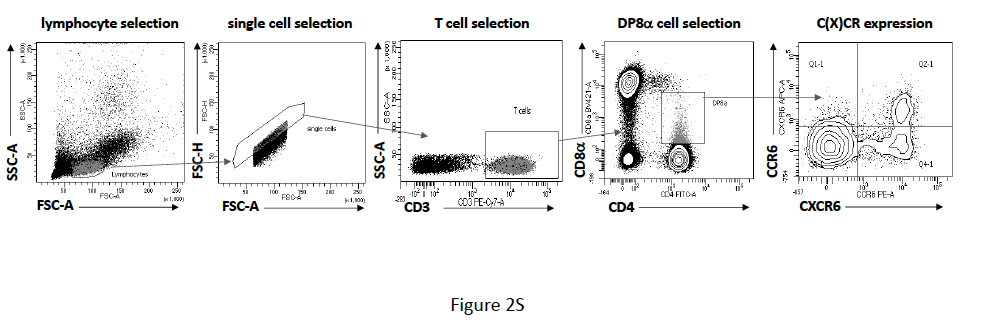


**Figure 2S:** Flow cytometry gating strategy for quantification of DP8α Treg cells in human blood samples.


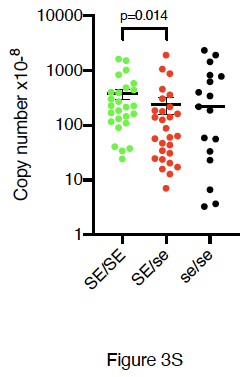


**Figure 3S:** Relationship between the *FUT2* status and the absolute quantification of *Faecalibacterium prausnitzii* in feces determined by qPCR, as described in the methods section. Two-sided Mann-Whitney test was used for comparisons. SE/SE=*FUT2* homozygote wild-type; SE/se=*FUT2* heterozygotes; se/se=*FUT2* null homozygotes.
